# Management of an advanced case of basal cell carcinoma in a patient with albinism: Considerations for working in low- and middle-income countries

**DOI:** 10.1016/j.jdcr.2024.12.012

**Published:** 2024-12-24

**Authors:** Chinecherem M. Chime-Eze, Simon Peter Mundeli, Julian Katungi, Aloyo Gladys Onguti, Grace Mulyowa

**Affiliations:** a Department of Pediatrics, University of North Carolina - Chapel Hill, Chapel Hill, North Carolina; b Department of Dermatology, Mbarara University of Science and Technology, Mbarara, Uganda

**Keywords:** 5-fluorouracil, basal cell carcinoma (BCC), carbon dioxide ablation laser therapy, excision, low- and middle-income countries (LMICs), oculocutaneous albinism (OCA)

## Introduction

Oculocutaneous albinism (OCA) is a disorder of melanin biosynthesis that results in hypopigmentation of the eye, skin, and hair.^1^ The most common cancers seen in individuals with albinism are basal cell carcinomas (BCCs) and squamous cell carcinomas.^1^ Historically, people with albinism had been thought to be more likely to develop squamous cell carcinomas. However, recent evidence has highlighted an increase in the occurrence of BCCs in this population.^2^ Although it remains unclear which cancer is dominant for this population, it is thought that the long indolent course of BCC leads to underdiagnosis and late presentation. In low- and middle-income countries (LMICs) where there are limited dermatologists, late presentation is unfortunately a common occurrence. In this report, we highlight the case of a Ugandan patient with OCA with delayed presentation of an advanced BCC of the face and the challenges of management in a low-resource setting.

## Case presentation

A 20-year-old woman from southwestern Uganda with type 2 OCA presented to the general dermatology clinic at the regional referral hospital with a 1-year history of a left cheek wound that was progressively increasing in size and associated with discharge and mild pain. The wound began as a nonpainful bump that later ulcerated. She gradually developed similar lesions on the right side of the forehead, right cheek, and right side of the neck as well as bilateral elbows over the course of the year. She had no improvement following a course of ampicillin and cloxacillin prescribed by a local health center nurse. Her albinism had been managed via sun avoidance without sunscreen. Because of the socioeconomic factors described later, she had never received previous care from a dermatologist or an ophthalmologist. She reported no other known chronic illnesses.

**Fig 1:**
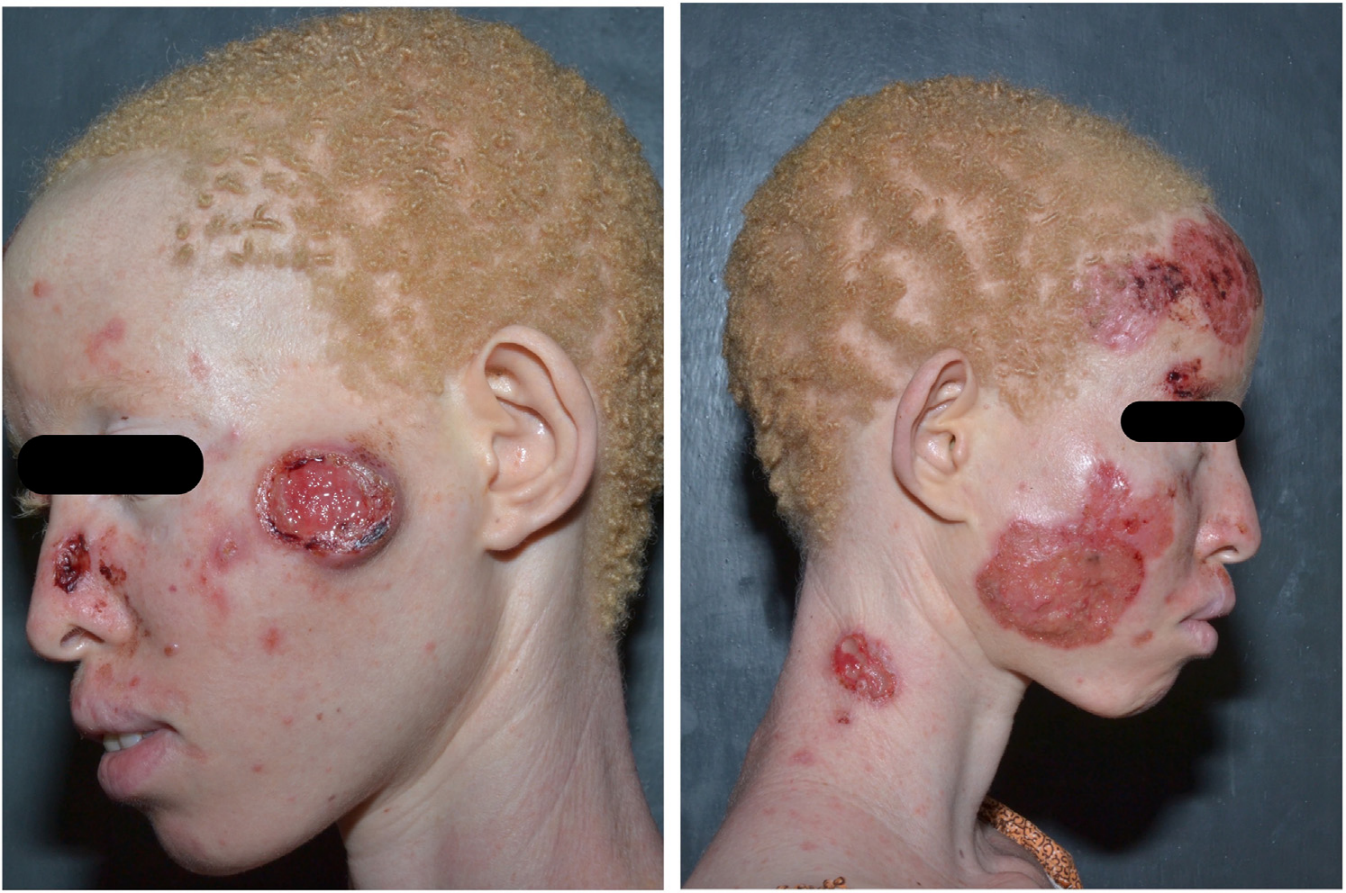
Basal cell carcinoma: initial presentation to dermatology clinic. Ulcerated lesions to the left zygomatic region of the face, right upper forehead, right medial and lateral cheek, and right superior side of the neck.

**Fig 2:**
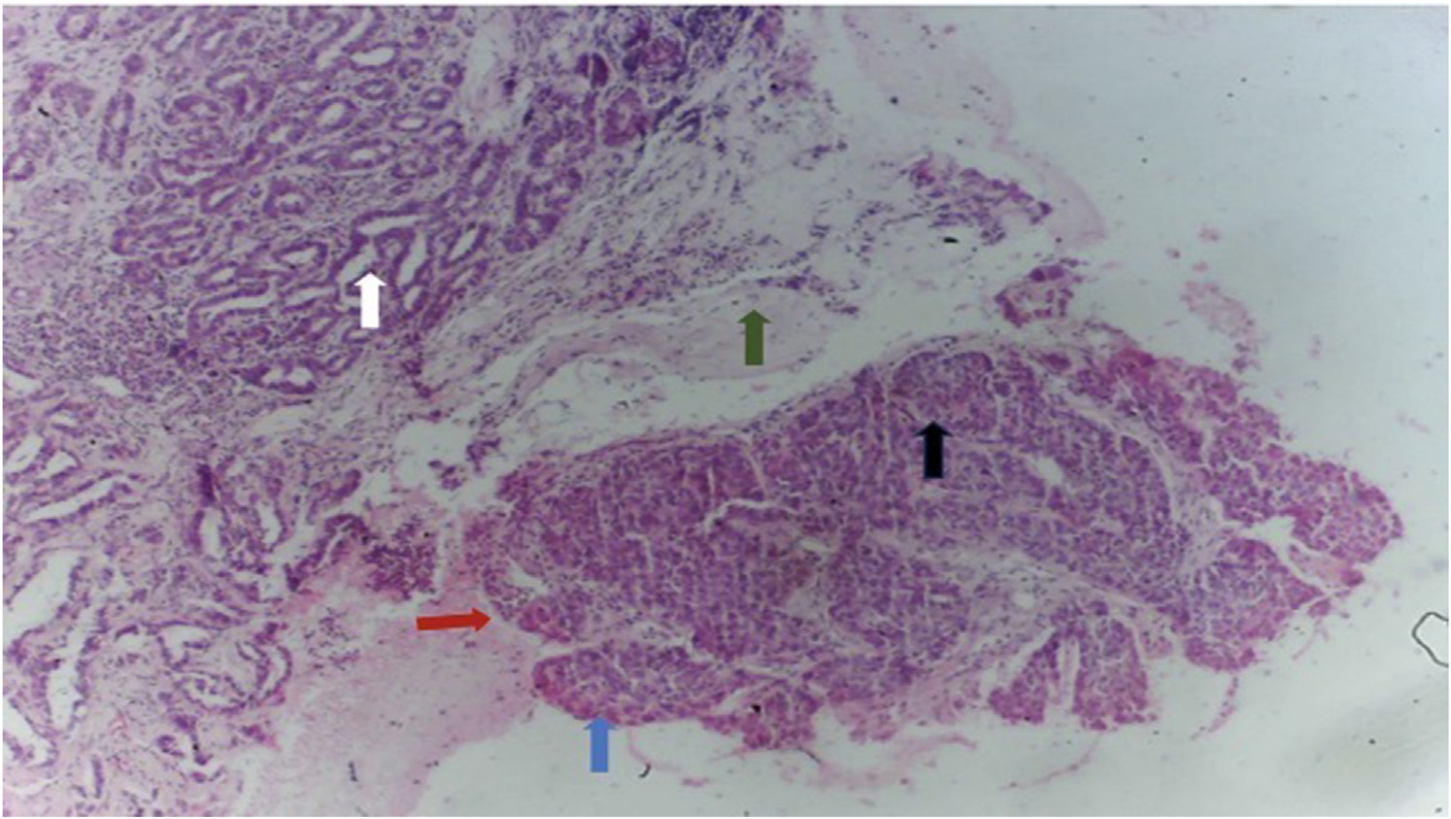
Basal cell carcinoma: photomicrograph of retraction artifact (*red arrow*), peripheral palisade (*black arrow*), eccrine differentiation (*white arrow*), sheets of basaloid epithelial cells (*blue arrow*), fibro-myxoid stroma (*green arrow*; hematoxylin-eosin, magnification ×4).

On examination, she had multiple ulcerated plaques with necrotic, erythematous centers with crusts and well-circumscribed elevated borders (rolled edges) located on the forehead, right cheek, left side of the nasal bridge, neck, elbows, and left zygomatic region concerning for BCC in the setting of OCA. She also had multiple skin-colored nodules on the face and side of the neck (Fig 1). An 8-mm punch biopsy was taken from an ulcer on the left side of the face. Histology revealed surface ulceration and infiltration by epithelial cells with high nuclear-to-cytoplasmic ratio, and minimal cytoplasm with high tumor cells forming cords with some evidence of palisading and eccrine differentiation (Fig 2). This confirmed the diagnosis of BCC with eccrine differentiation.

**Fig 3:**
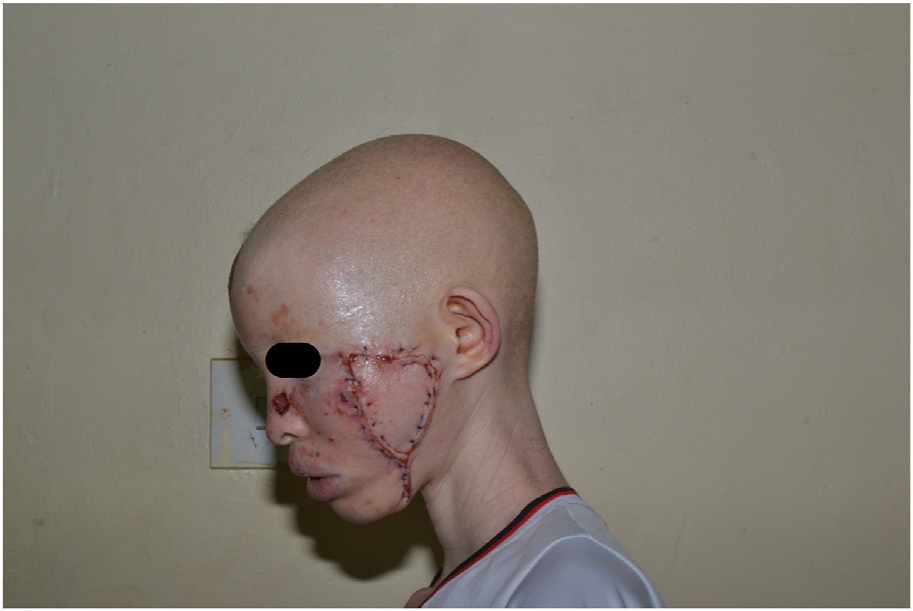
BCC: status post excision of left zygomatic BCC tumor and flap reconstruction. *BCC*, Basal cell carcinoma.

**Fig 4:**
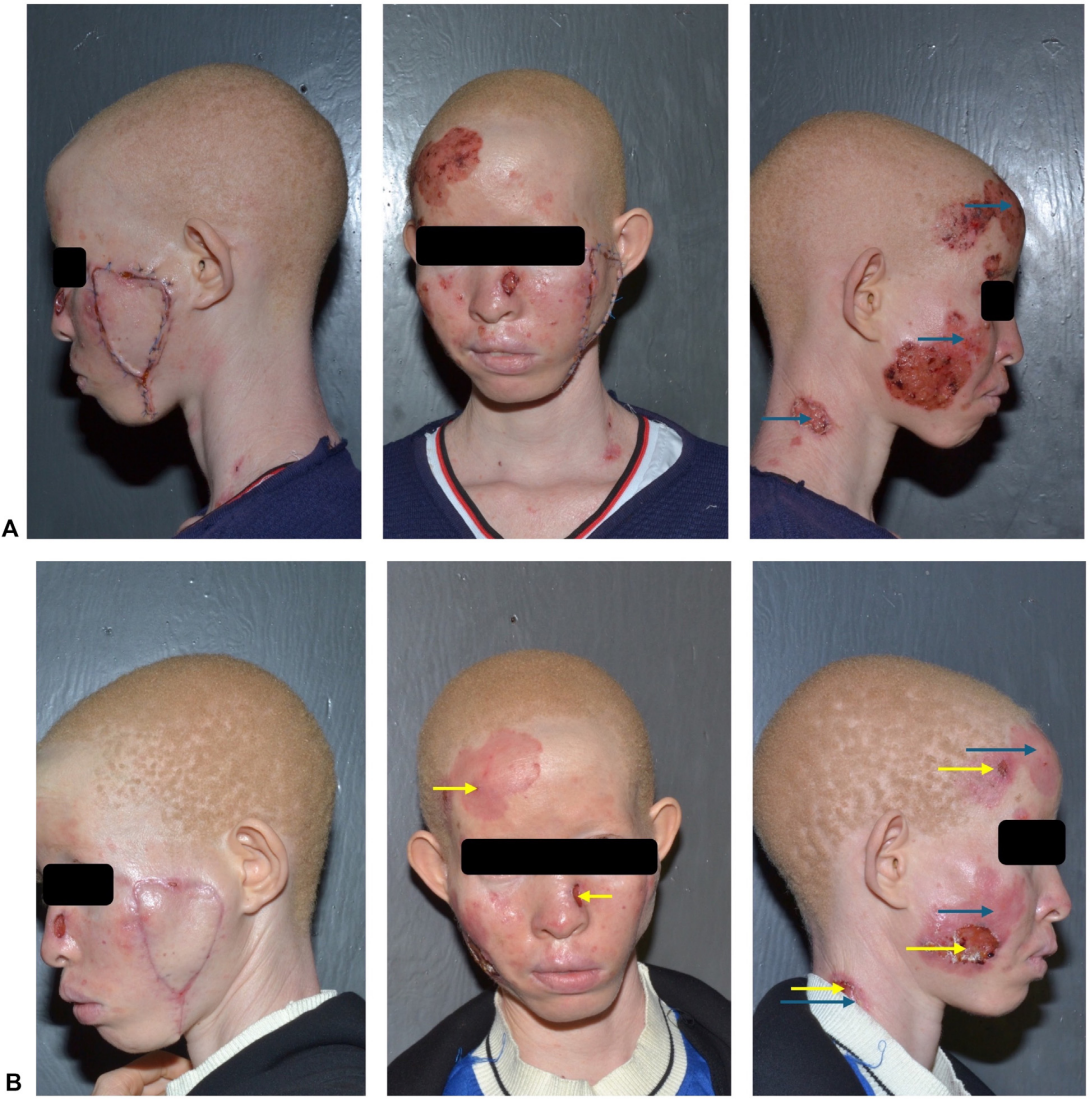
**A,** BCC: Six weeks after surgery with 5-FU application (*green arrows)*. **B,** BCC: 8 weeks postsurgery following carbon dioxide ablation laser therapy, 5-FU application (*green arrows*), and application of mupirocin ointment (*yellow arrows*). *BCC*, Basal cell carcinoma.

Management constituted surgical excision of the large, ulcerated nodule on the left cheek with flap reconstruction (Fig 3) by plastic surgery. Two weeks later, she underwent 3 sessions of carbon dioxide ablation laser therapy (continuous wave with a wavelength of 10,600 nm, fluence of 8 J/cm^2^, and a pulse width of 1 ms) 2 weeks apart for the remaining facial lesions. During this 6-week period, 5-fluorouracil (5-FU) cream was also applied to the lesions as an adjunct therapy (Fig 4, *A*). This was followed by application of mupirocin ointment twice daily for 2 weeks. She was noted on her follow-up visit to have reepithelialization on all facial lesions except the right cheek and left side of the nasal bridge while the flap was healing well (Fig 4, *B*). She was restarted on 5-FU cream for the persistent lesions on the right cheek and left side of the nasal bridge and continues to follow up with our clinic every 2 weeks. Sun protection measures and sunscreen were also recommended. Five months later, her left cheek surgical site is healed, whereas the right cheek lesions have progressed and are being considered for surgical excision.

## Discussion

Skin cancers are associated with OCA.^1^ Our patient’s course was complicated by delayed presentation, lack of access to care, and lack of proper treatment due to socioeconomic constraints, resulting in the progression of multiple BCCs on her face and neck.

Because the child of a single parent with limited resources, she had not visited an ophthalmologist or dermatologist before presenting to our clinic. Limited access to health care providers with proper training in diagnosing and managing skin disease results in seeking alternative treatment options. In some situations, some patients self-medicate without ever being evaluated by a clinician. In our patient’s case, she received an antibiotic prescription from a “drug shop” in her village run by a nurse, further delaying appropriate treatment.

Uganda’s health care system is structured in ascending order of capability—health centers, general hospitals, regional referral hospitals, and national referral hospitals. Specialists such as dermatologists are limited to a few regional referral hospitals and the national referral hospital.^3^ This hospital is the only facility offering a specialized training in dermatology in the entire country. This status affords access to foreign donations such as the CO_2_ laser used in the treatment of this patient, which is the only one available in the country. With the limited number of dermatologists in the country, however, she likely would have still faced difficulty accessing dermatologic care. Although a 2005 study reported a total of 6 dermatologists serving a population of over 25 million,^4^ anecdotal reports suggest that this number has not significantly changed with an estimated 11 dermatologists in 2024 serving a population of over 45 million.

Following initial treatment, our patient required multiple follow-up visits. She traveled 150 kilometers each way for each hospital visit using either a commercial motorcycle or taxi while incurring significant out-of-pocket costs. Prior studies have highlighted travel time as a barrier to seeking care among Ugandan dermatology patients.^5^

In addition to the financial limitations, cultural and social discrimination against persons with albinism further limits access to care. Dangerous beliefs regarding albinism include it being contagious or associated with superpowers can lead to life-threatening situations.^6^ Our patient expressed low self-esteem likely from consistent marginalization and cultural misunderstanding regarding her skin color.

This case highlights the importance of early detection and prevention of skin cancer in high-risk populations such as patients with albinism. Given the limited number of dermatologists in this area, it also illustrates the numerous challenges faced by patients with albinism in LMICs when accessing care. It illustrated the need to use initiatives such as task-sharing models that teach providers, such as health center nurses, who are often the first point of contact for patients to recognize life-threatening dermatologic conditions. This will allow for earlier diagnosis and intervention, which are critical in LMICs, where there are limited resources to manage advanced conditions. We hope that by reporting this case, we can raise awareness of the complexities and variations in clinical presentations and advocate for interventions that will address the needs of all patients including those with limited resources.

## Conflict of interest

None disclosed.
